# Assessment of the Peradeniya Organophosphorus Poisoning Scale as a Severity and Prognostic Marker in Patients With Acute Organophosphorus Poisoning Presenting to an Emergency Medicine Department

**DOI:** 10.7759/cureus.40277

**Published:** 2023-06-12

**Authors:** Nimesh B Malaviya, Rina Parikh, Krunalkumar Pancholi, O.B. Belim

**Affiliations:** 1 Emergency Medicine, Sir Sayajirao General (SSG) Hospital and Medical College, Baroda, Vadodara, IND

**Keywords:** mortality, ventilator, icu, atropine, peradeniya organophosphorus scale, op poisoning

## Abstract

Background

Organophosphorus (OP) compound poisoning is the most common toxicological medical emergency in India, where the majority of the population lives on agriculture. The Peradeniya Organophosphorus Poisoning (POP) scale can be a simple and effective system to determine the need for ventilatory support early in the course of admission. The objective of this study was to evaluate the prognostic value of the clinical parameters of the POP scale in predicting the severity of organophosphorus compound poisoning, by comparing early predicted patient prognosis evaluated by the POP scale on admission with the patient outcome.

Methods

This was a prospective observational study of acute organophosphorus compound poisoning presenting to the emergency department of Sir Sayajirao General (SSG) Hospital and Medical College, Baroda. We included patients over 12 years of age with a history of, or symptoms suggestive of, acute OP poisoning. The patients received initial resuscitation according to airway, breathing, circulation, disability, and exposure simultaneously with decontamination and gastric lavage by Ryle’s tube. They also received the standard antidotes of atropine and pralidoxime immediately. We applied the POP scale to each patient upon admission and graded the poisoning severity as mild (a POP scale score of 0-3), moderate (4-7), or severe (8-11). This scale assessed the patients’ need for mechanical ventilation and ICU management and their final clinical outcome.

Results

We enrolled 60 patients in the study. Most of them were under 20 years of age, and 65% of them were male. Social laborers were the major population, and most of them had suicidal intention. Monocrotophos was the most commonly consumed OP compound. Most of the patients were brought to the hospital within two to six hours of consumption. Vomiting and profuse secretions were the primary presenting symptoms. A majority of the patients (47) fell into the mild POP scale range. None of the patients had severe poisoning. Out of 60 patients, 49 patients improved and 11 patients died. Seven patients (15%) with mild POP scale scores and four patients (31%) with moderate scores died. Overall, 61.7% of patients with mild POP scale scores and 100% of patients with moderate scores needed mechanical ventilation.

Conclusion

The POP scale is an effective tool to measure severity and make a prognosis in patients with acute OP compound exposure. It may be a simple, inexpensive tool that may help predict the need for ventilatory support at admission. Early identification of danger signs may help in the reduction of mortality and morbidity when resources are limited. However, we found incorporating other clinical parameters and biochemical markers provides better prognostication than using the POP scale alone.

## Introduction

Organophosphorus (OP) compounds, namely, monocrotophos, malathion, parathion, chlorpyrifos, etc., have been widely used in agriculture for crop protection and pest control and are the most commonly used drugs for suicide attempts in India. The use of these products for deliberate self-harm has increased proportionately with their use in agriculture. In majority of the cases, the most common mode of poisoning is ingestion. Parasympathetic symptoms like excessive salivation, urination, spontaneous defecation, lacrimation, excessive vomiting and perspiration are seen in majority of cases. Important signs include miosis, bradycardia, bronchospasm, bronchorrhea and inadequate ventilatory efforts. Many patients require endotracheal intubation, invasive ventilatory support and intensive care, but managing all OP patients in the ICU is not possible because of a scarcity of resources in India.

Senanayake et al. developed the Peradeniya Organophosphorus Poisoning (POP) scale at the University of Peradeniya, Sri Lanka [1]. This may be a simple and effective system to determine the need for ventilatory support early in the course of admission. It helps identify high-risk patients early, allowing timely intervention. The usefulness of this scoring system based on clinical parameters is likely to be greater in developing countries like India.

## Materials and methods

This was a prospective observational study of 60 patients with acute organophosphorus poisoning presenting to the emergency ward of Sir Sayajirao General (SSG) Hospital and Medical College, Baroda. We performed the study over a one-year period from March 2020 to March 2021. We obtained approval (IECBHR/99-2020) from the Institutional Ethics Committee for Biomedical and Health Research, Medical College and SSG Hospital, Baroda, for the study.

The inclusion criteria were patient age over 12 years and presenting with a history of, or symptoms suggestive of, acute organophosphorus poisoning within 24 hours of consumption. We excluded patients who had definitely received atropine and/or pralidoxime outside the hospital. Informed consent was taken before starting the study on patients. A provisional diagnosis of OP toxicity was made on the basis of the definite history of poisoning given either by the patient or the patient’s relatives, which was further confirmed by the examination of the bottle or typical toxidrome comprising salivation, lacrimation, urination, defecation and gastric emesis (SLUDGE) along with fasciculations, miosis or characteristic odor of the compound.

Initial resuscitation

All patients fulfilling the inclusion criteria who presented to the emergency department (ED) were resuscitated according to airway, breathing, circulation, disability and exposure simultaneously. Decontamination and gastric lavage was done by Ryle’s tube. The standard antidote atropine 3 mg IV bolus with subsequent double doses every five minutes till signs of atropinization developed followed by continuous atropine infusion and pralidoxime 2 gm IV loading dose followed by IV infusion (6 gm Pam in 500 ml 5% dextrose) were given as initial management.

Peradeniya Organophosphorus Poisoning scale

We applied the POP scale, as shown in Table 1, to each patient at admission and graded their poisoning severity as mild (a score of 0-3), moderate (4-7), or severe (8-11). According to the scale severity, we prioritized patients for ICU admission and treatment. We closely followed up with and monitored all the patients in wards and ICUs. We considered patients with persistent cyanosis, low SpO_2_, apnea, hypoventilation, and/or persistent tachypnoea for timely intubation and ventilatory support.

**Table 1:** Peradeniya Organophosphorus Poisoning (POP) scale 0-3: mild poisoning, 4-7: moderate poisoning, 8-11: severe poisoning

Parameter	Criteria	Score
Pupil size	≥2 mm	0
	<2 mm	1
	Pinpoint	2
Respiratory rate	<20/min	0
	≥20/min	1
	≥20/min with central cyanosis	2
Heart rate	>60/min	0
	41-60/min	1
	<40/min	2
Fasciculation	None	0
	Present, generalized/continuous	1
	Both generalized and continuous	2
Level of consciousness	Conscious and rational	0
	Impaired response to verbal command	1
	No response to verbal command	2
Seizures	Absent	0
	Present	1

## Results

**Figure 1:**
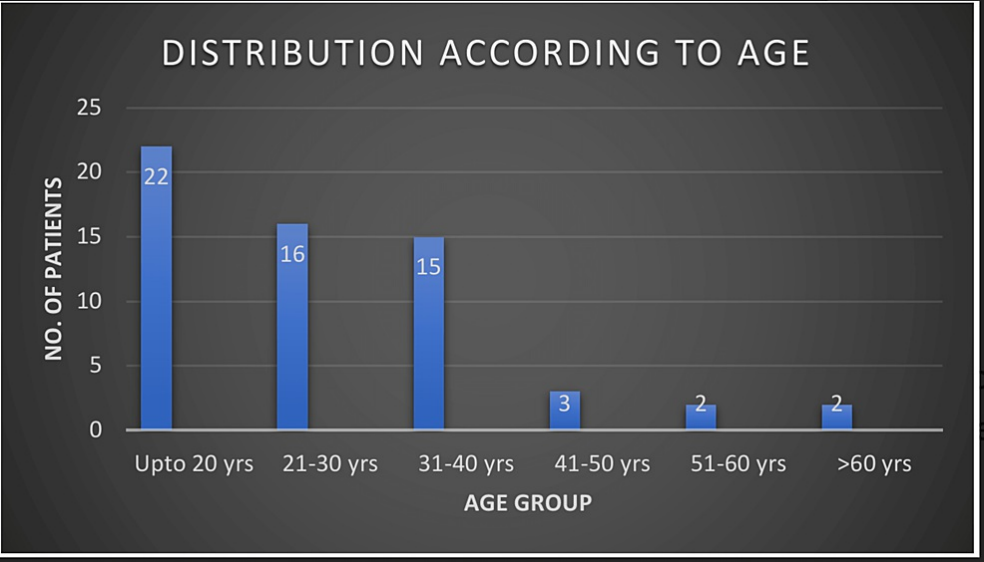
Patient distribution according to age

**Figure 2:**
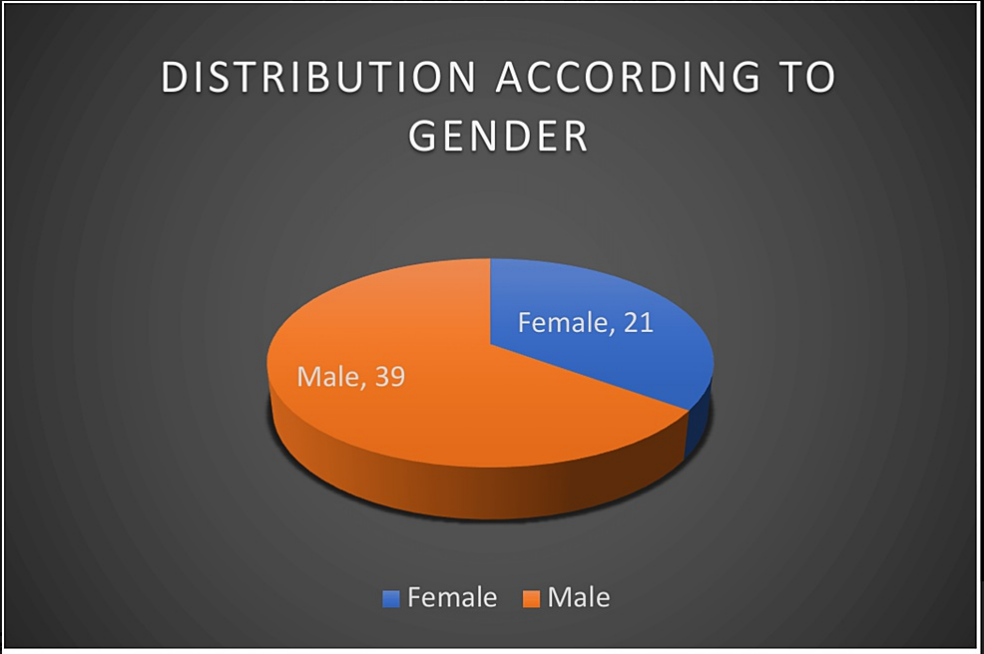
Patient distribution according to gender

**Figure 3:**
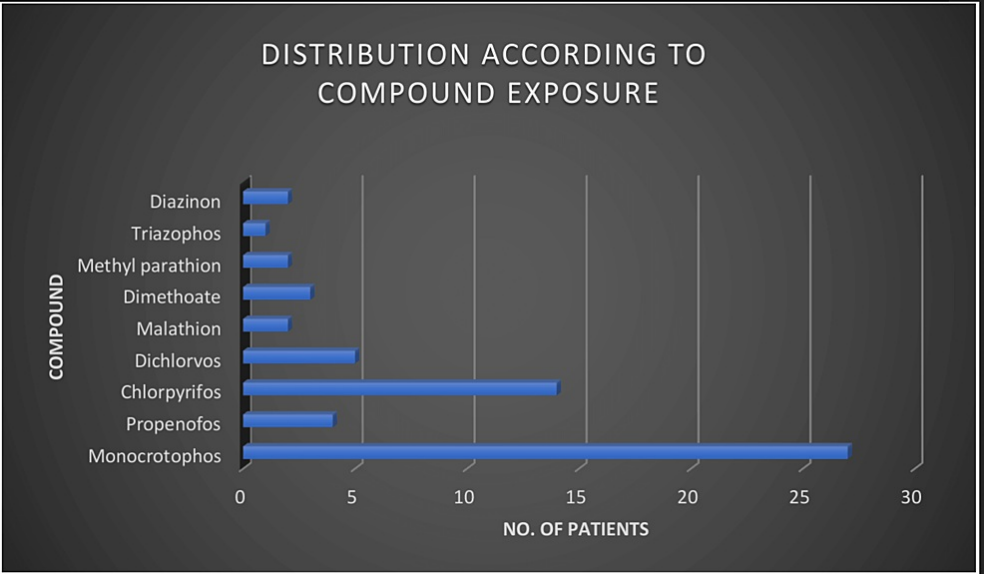
Patient distribution according to organophosphorus compound exposure

**Figure 4:**
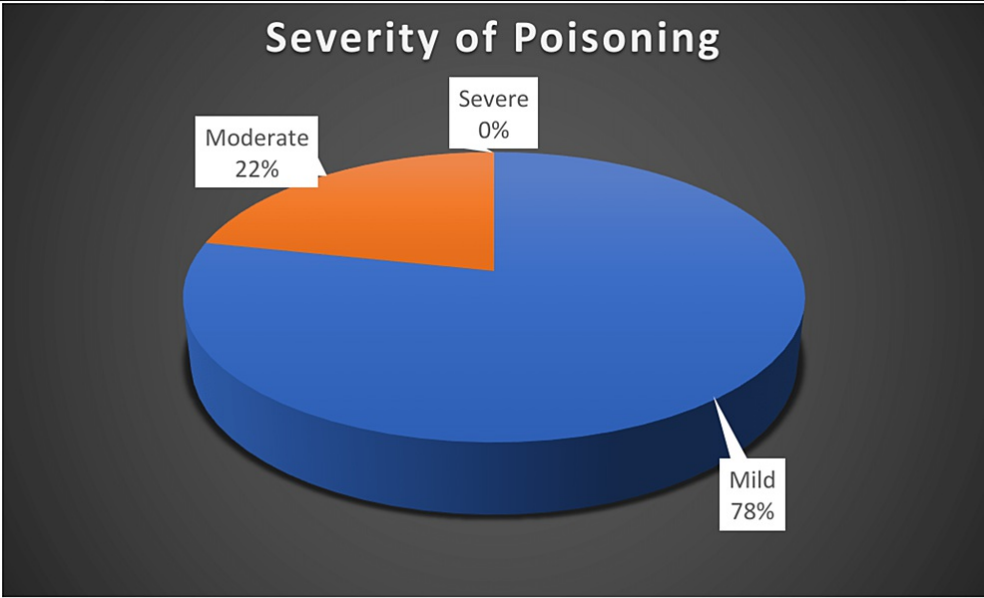
Patient distribution according to Peradeniya Organophosphorus Poisoning scale severity

We enrolled a total of 60 patients in the study (Table 2). Most of them were below 20 years of age; 65% (39) of them were male (Figures 1, 2, respectively). Occupationally, the majority of patients consisted of laborers, followed by farmers. Most patients were found to have a suicidal intention. Monocrotophos was the most commonly consumed OP compound, followed by chlorpyrifos. Other OP compounds were dichlorvos, propenofos, malathion, dimethoate, methyl parathion, triazophos, and diazinon (Figure 3). Most patients presented within two to six hours of OP compound consumption. Symptomatic vomiting and excessive secretion were present in a majority of patients. Other symptoms included breathlessness, loss of consciousness, diarrhea, and seizures. Table 3 and Figure 4 show that a majority of patients (47) were in the mild POP scale score range, 13 patients had moderate POP scale scores, and none of the patients had severe-grade poisoning.

**Table 2:** Study participants OP, organophosphorus

		n
	Total patients who came with OP poisoning during the study period	72
Excluded patients	Already taken atropine and/or pralidoxime from outside before presentation	10
	Refused admission	2
Included patients	Total patients included in the study	60

**Table 3:** Patient distribution according to the POP scale POP scale, Peradeniya Organophosphorus Poisoning scale

POP scale	No. of patients	Need for ventilatory support	Survival rate, % (n)	Patients requiring ICU stay
Mild (0-3)	47	29	85.1% (40)	33
Moderate (4-7)	13	13	69.2% (9)	13
Severe (8-11)	0	0	-	-

Overall, 70% of patients (42) required ventilatory support, including 61.7% patients (29) with mild POP scale scores and 100% patients (13) with moderate scores. Patients with late presentation to the hospital after consumption had increased ventilatory requirements with more severe respiratory depression. All patients who had consumed propenofos, dimethoate, methyl parathion, and triazophos required mechanical ventilation.

Among the 60 patients, 82% (49) improved, 11 patients expired due to complications, and 85.1% of patients with mild poisoning improved. The mortality rate was 30.8% for patients with moderate poisoning. Nine out of 10 patients who presented to the hospital within two hours of OP exposure survived. A total of 42 patients required ventilator support, of whom 31 patients survived (74%) and 11 patients died (26%). All 18 patients who did not require ventilator support survived.

## Discussion

Organophosphorus poisoning is a common health hazard in developing countries. Some patients with OP poisoning may require ventilator support and intensive care due to respiratory failure, but an intensive care facility may not be readily available because of limited resources and a high patient load. Therefore, the segregation of patients with a high risk of death is helpful in reducing mortality. We performed this study to assess the prognostic value of such clinical parameters in predicting clinical outcomes in OP poisoning.

Most of the patients were under 20 years of age (36.66%), followed by 26.66% of patients in the age group of 21-30. Studies by Barik, Tripathi, and Raikod et al. have shown that OP compound poisoning is common in the age group of 21-30 (Figure 3) [2-4]. Most of the patients in our study population (65%) were male; the male-to-female ratio was 1.85:1. A similar male preponderance was observed by Kamath and Gautam and other researchers in their studies [2,3,5-7].

Our study revealed that 88% of patients consumed poison with a suicidal intention, and only 12% were accidental exposures. These values correlate with observations made by Prakash et al. and also other researchers [3-5,8-10]. Figure 4 shows the most commonly used OP compounds for poisoning. The most common compound was monocrotophos (45%), followed by chlorpyrifos (23.33%) and dichlorvos (8.33%). The study by Prakash et al. also showed that the most common compound consumed was monocrotophos [5,8]. The compounds differed according to region and local use. Vomiting and excessive secretion were symptoms in a majority of patients [5]. Other symptoms included breathlessness, loss of consciousness, diarrhea, and seizures.

In our study, 78.33% of the patients had mild poisoning according to the POP scale, 21.66% of patients had moderate poisoning, and no patients had severe poisoning. Vernekar and Shivaraj showed 50% of patients with mild poisoning, 44% of patients with moderate poisoning, and only 6% of patients with severe poisoning according to the POP scale [11]. Study results of Mevada et al., Raveendra et al. and Yaduraj et al. also correlate closely with our study [7,10,12]. A study by Barik showed mild poisoning in 40% of patients, moderate in 45.3%, and severe in 14.7% [2].

In our study, 70% (n=42) of the patients required ventilatory support; respiratory failure primarily motivated intubation. The criteria for intubation were a respiratory rate of <10/min, inadequate chest rise suggestive of impaired self-respiratory efforts, thoraco-abdominal breathing asynchrony, and failure of non-invasive airway measures to maintain adequate oxygen saturation. In a study by Kavya et al., 80% of patients required ventilatory support, which was comparable to the results of our study [13].

Overall, 29 patients (61.7%) with mild poisoning required ventilator support, and all 13 patients with moderate poisoning (100%) needed ventilator support. The reasons behind the higher number of patients with mild poisoning requiring ventilatory support were as follows: (1) majority of patients presented with respiratory failure irrespective of the presence or absence of other parameters of the POP scale and (2) a higher number of patients were diagnosed with mild poisoning. When we compared individual parameters of the POP scale, such as pupil size, respiratory rate, heart rate, fasciculations, level of consciousness, and seizures, all except heart rate, fasciculations, and seizures were significant in requiring ventilator support. A study by Shabari et al. showed that among patients, 14.8% with mild, 30% with moderate, and 46.1% with severe poisoning required ventilatory support [14]. According to our findings, ventilatory failure requires special consideration and incorporation to assess the severity of OP poisoning in addition to using the POP scale.

Out of 60 patients, 49 (81.66%) improved and 11 (18.33%) died. This data correlates with the findings of Mevada et al. [7]. The most common reason for mortality in OP poisoning patients was respiratory failure; 82% of patients died due to acute respiratory failure, followed by hypoxic brain injury, whereas only two patients died due to secondary infections like ventilator-associated pneumonia, sepsis, and multi-organ dysfunction syndrome. Another limitation was the difficulty in obtaining ICU beds for mechanical ventilation because of our limited resources and the COVID-19 crisis. A study by Raikod et al. showed an 82% survival rate, while Tripathi showed 82.5% and Sindhu et al. showed 83% survival [3,4,15]. These findings are comparable to those of our study.

In our study, only 16.66% of patients presented within two hours of consumption of OP compounds, while 6.66% of patients presented after 12 hours. A majority of patients (31 out of 60, 51.66%) presented within two to six hours. This delayed presentation may be due to the lack of awareness about a prompt visit to a health facility, which is vital. Some patients might have used home remedies, exhibited a late onset of symptoms, traveled from remote areas, lacked access to transportation, or were referred from a primary health center (PHC) or community health center (CHC), where they were admitted for a period of time. Prakash et al. showed that 58% of patients reached the hospital within four hours, while 42% of patients reached the hospital after four hours [8]. In our study, only one out of 10 patients died who presented within two hours of OP exposure. Mortality increased as the delay in hospital presentation increased; the mortality rate was 23% when patients presented within two to six hours and 20% when patients presented within 6 to 12 hours. The fact that no patients died who presented 12 hours after OP exposure might be because the patients consumed a mildly toxic OP compound or consumed only a small quantity insufficient for severe toxicity. This result might also be due to the small sample size of only four patients. Mortality was 26% in patients requiring ventilatory support, and 74% patients survived in our study.

A total of 33 out of 47 patients (70.2%) with mild POP severity required ICU care for mechanical ventilation or close observation, whereas all 13 patients (100%) with moderate POP severity required an ICU stay. The mean duration of ICU stays was 5.82 days for mild poisoning and 9.67 days for moderate poisoning. Regmi et al. had similar findings [6].

We encountered some limitations while performing this study. Because of the COVID-19 pandemic and the resulting nationwide lockdown during the study period, the sample size we obtained was relatively small due to non-availability of OP compounds, patients’ restriction to containment zones, and limited availability of public transport services; hence, our data calculation and findings could not be accurate. Serial monitoring and POP scoring were not possible for all patients as they were transferred to other settings (ICU and ward) immediately after stabilization. Because many patients were referred from a PHC and CHC with inadequate documentation of the drugs given, some parameters in the POP scale like pupil size and heart rate were biased due to possible atropine administration outside. Due to the busy environment of the emergency department, fasciculation could not be appreciated properly or might not have been present at the time of evaluation. We might not have considered seizures prior to ED presentation because relatives could not confirm them to be true seizures or hypoxia-induced rigidity. Relatives might not have witnessed seizures because they found the patient already in an altered sensory state; therefore, symptoms occurring between the onset of symptoms and relatives’ discovery of the patient might be unknown. We performed this study with a relatively small number of patients with limited parameters, so further studies with different parameters and larger populations may be necessary to validate the accuracy of our study.

## Conclusions

The POP scale is effective in assessing severity and making a prognosis in patients with acute OP compound exposure. When used at presentation in acute OP poisoning patients, the POP scale is an easy, inexpensive tool that may assist in predicting the need for ventilatory support. Early identification of danger signs may help in the reduction of mortality and morbidity in a resource-limited environment. We found it necessary to incorporate other clinical parameters (single breath count, use of bedside spirometry, presence of gurgling sound and pulse oximetry) as well as biochemical markers (PaO_2_ and PaCO_2_) to achieve a better prognosis.
